# Mediterranean-Type Diet and Brain Structural Change from 73 to 79 Years in the Lothian Birth Cohort 1936

**DOI:** 10.1007/s12603-022-1760-5

**Published:** 2022-03-17

**Authors:** Michelle Luciano, J. Corley, M.C.Valdés Hernández, L.C.A. Craig, G. McNeill, M.E. Bastin, I.J. Deary, S.R. Cox, J.M. Wardlaw

**Affiliations:** 1 Department of Psychology, University of Edinburgh, 7 George Square, EH9 8JZ, Edinburgh, UK; 2 Neuroimaging Sciences, Centre for Clinical Brain Sciences, University of Edinburgh, Edinburgh, UK; 3 Scottish Imaging Network, A Platform for Scientific Excellence (SINAPSE) Collaboration, Department of Neuroimaging Sciences, University of Edinburgh, Edinburgh, UK; 4 UK Dementia Research Institute Centre at the University of Edinburgh, Edinburgh, UK; 5 Institute of Applied Health Sciences, University of Aberdeen, Aberdeen, UK; 6 Global Academy of Agriculture and Food Security, University of Edinburgh, Edinburgh, UK

**Keywords:** Brain atrophy, brain volume, longitudinal study, trajectory model

## Abstract

**Objectives:**

To test whether Mediterranean-type Diet (MeDi) at age 70 years is associated with longitudinal trajectories of total brain MRI volume over a six-year period from age 73 to 79.

**Design:**

Cohort study which uses a correlational design.

**Setting:**

Participants residing in the Lothian region of Scotland and living independently in the community.

**Participants:**

A relatively healthy Scottish sample drawn from the Lothian Birth Cohort 1936.

**Measurements:**

Total brain volume measurements were available at ages 73, 76 and 79 (N ranged 332 to 563). Adherence to the MeDi was based on food frequency questionnaire data collected three years before the baseline imaging scans, and was used in growth curve models to predict the trajectory of total brain volume change.

**Results:**

No association was found (p>.05) between adherence to the MeDi at age 70 and total brain volume change from 73 to 79 years in minimally-adjusted (sex) or fully adjusted models controlling for additional health confounders.

**Conclusions:**

Variation in adherence to the MeDi was not predictive of total brain atrophy over a six-year period. This suggests that previous findings of dietary associations with brain volume are not long lasting or become less important as ageing-related conditions account for greater variation in brain volume change. More frequent collection of dietary intake data is needed to clarify these findings.

## Introduction

Eating a Mediterranean-type diet (MeDi) has benefits on physical and mental health (1, 2, 3, 4, 5) that may be particularly relevant to healthy ageing (6). This diet is characterised by high consumption of the following foods: fruit, vegetables, legumes and cereals; and low consumption of red meat and poultry. A moderate intake of fish, and low to moderate intake of dairy products and wine (with meals), plus olive oil as the foremost fat source, is also typical of this diet. Identifying the potential biological mediators of the effect of MeDi diet on cognitive function and dementia can help us understand the mechanisms of action. One potential biological mediator is brain volume; that is, one might hypothesise that MeDi affects brain volume (protecting against atrophy), which in turn affects cognitive function (i.e., maintenance of function). In previous research (7), using the same cohort as that studied in the present report, we showed that increased adherence to the MeDi in a Scottish population, 401 members of the Lothian Birth Cohort 1936 (LBC1936), did not associate with level of total brain volume but did predict change in total brain volume over a three-year period, explaining .5% of variance. The present study assesses whether this association endured over a further three years.

In our prior study (7), we investigated total brain volume, grey matter volume and cortical thickness, but only total brain volume change was associated with the MeDi. Additionally, we did not find that higher fish and lower meat intake drove the MeDi associations with brain structural change despite cross-sectional evidence of this from the United States (8). Therefore, in the current study, we focus only on total brain volume and on overall MeDi. A third imaging wave data from the LBC1936 (79 years of age) enabled specification of a growth curve model, and whereas we did not expect MeDi associations with the intercept (brain volume base level), we expected that the association of MeDi with the slope (brain volume change) would persist over this longer period.

## Methods

### Participants

Participants were part of the LBC1936; most resided in the Edinburgh region of Scotland (N = 1091; 49.8% female) (9). All were relatively healthy, living independently and did not have a dementia diagnosis at recruitment. At their baseline examination between 2004 and 2007, they were around 70 years of age and completed cognitive tests and health surveys, which were repeated longitudinally at roughly 3-year intervals (10). Food frequency data (7) were collected in 967 participants at baseline by postal questionnaire: 124 participants did not return these or did not fill them in. Around three, six, and nine years later, those participants who were willing and able to continue with the study had a structural MRI brain scan. For those with food frequency data, volumetric data was available from 563 participants at age 73 years (72.69 ±0.72), 416 participants at 76 years (76.38 ±0.66), and 332 participants at 79 years (79.43 ±0.64).

### Standard Protocol Approvals, Registrations, and Patient Consents

Ethics permission was granted from the Multi-Centre Research Ethics Committee for Scotland (Wave 1:MREC/01/0/56), the Lothian Research Ethics Committee (Wave 1: LREC/2003/2/29), and the Scotland A Research Ethics Committee (Waves 2, 3, & 4: 07/MRE00/58).

### Mediterranean Diet (MeDi)

The MeDi score was based on data gathered from the Scottish Collaborative Group 168-item Food Frequency Questionnaire, version 7 (11, 12) as previously described (7). Medians (adjusted for caloric intake and sex) split low from high consumption for each component, with beneficial components (fruit, vegetables, legumes, cereal, fish, monounsaturated fatty acids to saturated fatty acids ratio) scoring greater than or equal to the median scored as 1, and detrimental components (meat, dairy) greater than or equal to median assigned a 0. Moderate alcohol consumption (for men between 10 to 50 g alcohol per day; for women between 5 and 25 g per day) was considered beneficial and scored as 1. The sum of component scores (ranging 0 to 9) represented adherence to the MeDi, with higher scores representing greater adherence.

### Covariates

#### Fixed

Total years in full-time education represented educational attainment. Measures of premorbid IQ and general cognitive ability were collected from baseline and might influence a person's dietary choices (13): pre-morbid IQ was estimated by the National Adult Reading Test (NART) (14) and general cognitive ability represented the first unrotated principal component from a range of cognitive tests (see (15)). The APOE e4 allele was coded as absent or present and used in sensitivity analysis. Genotyping was carried out by the Wellcome Trust Clinical Research Facility Genetics Core, Western General Hospital, Edinburgh using TaqMan® technology on genomic DNA isolated from whole blood.

#### Time varying

At each data wave, information on the following was collected: current cognitive impairment (Mini Mental Status Examination (MMSE) 16); body mass index (BMI) using clinical measurements of height and weight; and self-reported history of stroke, cardiovascular disease, high blood pressure and diabetes.

### MRI

A detailed description of the brain MRI procedure and image processing can be found in Wardlaw et al., (17). A GE Signa 1.5 T HDXT clinical scanner gathered structural T2-, T2*-, FLAIR- and T1-weight MRI data. Intracranial volume included tissue within the inner skull table (venous sinuses to the axial slice inferior to the inferior edge of the cerebellar tonsils and on/superior to the superior tip of the odontoid process).

#### Statistical Analysis

The effect of MeDi on longitudinal change in TBV was tested using a growth curve analysis in a structural equation modelling framework (lavaan package in R (18)), adjusting for covariates in a series of models and comparing models using standard goodness of fit statistics. Because of well-known issues with selective drop-out in ageing studies, we chose to use full information maximum likelihood estimation to reduce selection bias as has been done previously in this cohort (19). Slope weightings starting at 0 were set as the average interval in years between imaging test sessions, 3.7 and 6.76. The covariate selection was based on our previous work in this sample (7). Model 1 was minimally adjusted, including sex as a time invariant covariate. Model 2 additionally included years of education and pre-morbid IQ as time invariant covariates, and BMI, diabetes, high blood pressure, history of stroke, and cardiovascular disease history as time varying covariates. Model 3 included covariates that aligned with a large prior study of the MeDi and brain MRI (8): sex, education, baseline general cognitive ability (time invariant), age, BMI, diabetes, and MMSE as a possible cognitive impairment indicator (time varying). APOE e4 presence which can confound brain volume (20, 21), was controlled for in a sensitivity analysis.

To more closely replicate the analysis used in our previous work, but over a longer six-year period, we also ran a regression analysis where TBV at age 79 was the outcome measure but including TBV at age 73 as a covariate in the series of three models with varying covariates. This removed stable MRI variance across time from the outcome measure so that the residual reflected change in TBV over the six-year period and any unique measurement error.

## Results

TBV and covariate descriptive statistics are shown for the full sample and separately for groups at baseline with lower versus higher adherence to the MeDi (Table 1). These unadjusted variables did not differ between MeDi groups. Subsequent analyses use the continuous MeDi scale. Mean quantitative MeDi score between Wave 4 participants (n = 298) and those who dropped out of the study from baseline (n = 265) did not differ (0.15 difference; t =1.01, df = 560.73, p = 0.31). Demographic and health traits did not differ between MRI-completers and non-completers, with the exception of cognitive ability and educational attainment where scores were higher in the completers (results shown in supplementary Table e-1).

**Table 1:** Covariates and Total Brain Volume Grouped by Lower Versus Higher Adherence to the MeDi for Participants with Complete Data

	Total*	Lower MeDi (0–4)**	Higher MeDi (5–9)***	**P**^∼^
N	298	157	141	
Time invariant				
Education (mean, SD)	10.95, 1.15	10.99, 1.17	10.90, 1.14	.518
Female (n, %)	136, 45.6	76, 48.4	60, 42.5	.370
NART	35.96, 7.55	35.94, 7.38	35.99, 7.76	.955
APOE e4 (n, %)	81, 28.3 (n=286)	39, 26.3 (n=148)	42, 30.4 (n=138)	.526
Baseline Cognitive Ability	.31, .93 (n=294)	.30, .91 (n=153)	.32, .95	.849
Time varying				
*Age 73*				
Stroke (n, %)	19, 6.4	10, 6.4	9, 6.4	1
Diabetes (n, %)	20, 6.7	9, 5.7	11, 7.8	.631
Hypertension (n, %)	137, 46	74, 47.1	63, 44.7	.758
Cardiovascular disease (n, %)	78, 26.2	39, 24.8	39, 27.7	.674
BMI	27.4, 4.03	27.21, 4.22	27.63, 3.82	.367
MMSE	29.01, 1.12	28.95, 1.15	29.07, 1.07	.346
*Age 76*				
Stroke (n, %)	33, 11.1	17, 10.8	16, 11.3	1
Diabetes (n, %)	31, 10.4	14, 8.9	17, 12.1	.486
Hypertension (n, %)	158, 53	86, 54.8	72, 51.1	.560
Cardiovascular disease (n, %)	100, 33.6	52, 33.1	48, 34	.964
BMI	27.33, 4.05	27.18, 4.31	27.50, 3.74	.499
MMSE	28.76, 1.58	28.61, 1.90	28.91, 1.12	.090
Age 79				
Stroke (n, %)	43, 14.5 (296)	23, 14.8 (155)	20, 14.2	1
Diabetes (n, %)	31, 10.4	17, 10.8	17, 12.1	.880
Hypertension (n, %)	175, 58.7	93, 59.2	82, 58.2	.943
Cardiovascular disease (n, %)	106, 35.6	58, 36.9	48, 34	.688
BMI	27.12, 4.20	26.91, 4.43	27.35, 3.93	.361
MMSE	28.57, 2.2	28.43, 2.62	28.72, 1.60	.234
Total Brain Volume Ratio				
Age 73	.69, .02	.68, .02	.69, .02	.438
Age 76	.67, .02	.67, .02	.67, .02	.093
Age 79	.66, .02	.66, .03	.67, .02	.105


Note: Demographic and cognitive measures were those measured at baseline. BMI = body mass index, MMSE = mini mental state examination; ∼ p values estimated from χ^2^ for binary traits and independent group t-test for continuous traits.

Fit statistics for the growth curve models of TBV are shown in Table 2 and supported good model fit for all three models (e.g., RMSEA all < .05). All models showed significant variability in TBV at baseline (p < .0001) and in change over time (p < .05). There was no significant correlation between TBV intercept and slope. The MeDi was not significantly associated with either the intercept or slope in the baseline model nor in models 2 and 3 where further covariate adjustments were made (see Table 3); sensitivity analysis results were also null.

**Table 2:** Fit Statistics of growth curve model applied to total brain volume

**Model**	***χ*2 (df)**	**CFI**	**RMSEA (90% CI)**	**SRMR**
(1) Sex and ICV adjusted	9.616 (4), p = .047	.998	.049 (.0, .089)	.004
(2) + Health confounders	40.027 (36), p = .296	1	.017 (.0, .041)	.008
(3) + cognitive ability	46.363 (42), p = .297	1	.016 (.0, .039)	.008

**Table 3:** Standardised fixed effect of MeDi on intercept and slope values from growth curve modelling of total brain volume at ages 73, 76, and 79 years

**Model**	**Intercept (Level)**			**Slope (Change)**		
	**Estimate**	**SE**	**P**	**Estimate**	**SE**	**P**
(1) Sex and ICV adjusted (N = 595)	.023	.015	.126	.07	.091	.441
(2) + Health confounders (N = 396)	.021	.017	.219	.067	.09	.459
(3) + cognitive ability (N = 396)	.019	.017	.272	.073	.098	.458

Alternative multiple regression modelling, where TBV change from first to third MRI wave was considered, again showed no effect of the MeDi in minimally adjusted or more fully adjusted models (p > .05).

## Discussion

The present study aimed to extend our previous finding of a MeDi apparently protective association with brain ageing over a three-year interval (7) to a six-year interval. Growth curve modelling of TBV using three time-points (age 73, 76, and 79 years) and TBV change from age 73 to age 79 did not support an effect of the MeDi. To reconcile this finding with the protective effect reported over a three-year interval in this cohort, one might conclude that the protective effect of the MeDi is short-lived. The latter interpretation is consistent with the findings of contemporaneous associations between MeDi and brain imaging measures (22). In our study, dietary intake was measured two to three years before the first MRI measurement, and could predict brain atrophy almost six years later but not nine years later. Changes in diet over this nine-year period (which is more likely than over a shorter period of follow-up) could also explain the absence of an association over this length of time, although we were unable to directly confirm this. Alternative explanations might be that our previous finding was type 1 error or that physiological age-related factors become more pronounced with advancing age and thus override any beneficial effects of diet.

Besides our previous study (7) that showed a MeDi association with TBV change over a 3-year period, only one other study (23) has measured the association of the MeDi on brain imaging structural change (specifically grey mater volume) over time. No association of MeDi on grey matter volume was found in their sample of 70 middle-aged (30 to 60 years) adults measured across a minimum of two years. However, high vegetable intake, which is a component of the MeDi, has been related to less grey matter volume change over a four-year interval in Korean adults from the general population aged between 49 and 79 years (24). Clearly, this is an emerging research area and more studies are needed on these longitudinal relations to be able to draw any conclusions.

Our cohort design is optimal in several aspects. The homogenous ethnic background and small age range reduces confounding due to genetic/cultural factors and epoch effects, although generalisability of the findings are limited to a Scottish population. The dietary information pre-dates the imaging collection allowing prospective effects to be studied. MRI collection over multiple equally spaced intervals and up to six years later enables application of more sophisticated statistical models and the potential to observe greater brain change than shorter periods. The main limitation of our design and one that might influence the null finding we report is that the MeDi score was only available at baseline, so we cannot be certain that participants had adhered to the same diet over the nine-year period. If differences in diet (away from a MeDi) had occurred then this would suggest that the MeDi needs to be sustained for lasting effects (i.e., beyond 3 years) on brain health. If substantiated this could have very important population health implications, with a lifestyle change to the MeDi potentially protective against brain atrophy and any downstream effects of this. Future studies require longer and more frequent assessment of diet across the lifespan to enable modelling of participants who have adhered longer to the MeDi.

## Statistical Analysis

Conducted by Dr Michelle Luciano, University of Edinburgh.

## Search Terms

Cohort studies, MRI, risk factors in epidemiology.
