# Supplementary material for: Mediterranean-Type Diet and Brain Structural Change from 73 to 79 Years in the Lothian Birth Cohort 1936
Source: J Nutr Health Aging. 2022 Mar 17;26(4):368–72. doi: 10.1007/s12603-022-1760-5 (PMC12879200; doi:10.1007/s12603-022-1760-5)
Supplement: Supplementary file 1 — Appendix [file mmc1.docx]

Supplementary Table e-1. Baseline Demographic, Health, and Cognitive Profile Comparisons of Participants with MeDi data and first wave MRI who completed all MRI waves (Completers) versus those who dropped out (Non-completers).

|  | MRI Completers | MRI Non-completers | P˜ |
| --- | --- | --- | --- |
| N | 298 | 265 |  |
| **Demographics** |  |  |  |
| Education (mean, SD) | 10.95, 1.15 | 10.67, 1.04 | .003 |
| Female (n, %) | 136, 45.6 | 134, 50.6 | .278 |
| APOE e4 (n, %) | 81, 28.3 (n=286) | 74, 29.2 (n=253) | .887 |
| Stroke | 10, 3.4 | 11, 4.1 | .784 |
| Diabetes | 17, 5.7 | 22, 8.3 | .296 |
| Hypertension | 107, 35.9 | 105, 39.6 | .411 |
| Cardiovascular disease | 65, 21.8 | 58, 21.9 | 1 |
| Body Mass Index | 27.17, 3.78 | 27.86, 4.50 | .051 |
| **Cognitive** |  |  |  |
| NART | 35.96, 7.55 | 34.55, 7.92 | **.032** |
| Cognitive Ability | .31, .93 (n=294) | .03, .96 (n=260) | .**001** |
| MMSE | 28.99, 1.29 | 28.83, 1.36 | .153 |
| **MeDi** | 4.36, 1.85 | 4.51, 1.68 | .312 |

Note: NART = National Adult Reading Test; MMSE = mini mental state examination; ˜ P values estimated from χ² for binary traits and independent group t-test for continuous traits.
